# Roles of organic anion transporting polypeptides in hepatocellular carcinoma

**DOI:** 10.3389/fgene.2025.1550723

**Published:** 2025-07-15

**Authors:** Hubert Chen

**Affiliations:** College of Arts and Science, Vanderbilt University, Nashville, TN, United States

**Keywords:** hepatocellular carcinoma, organic anion transporter polypeptides, SLCO genes, survival, prognostic markers

## Abstract

**Introduction:**

Hepatocellular carcinoma (HCC) is the most common primary liver malignancy, predominantly occurring in patients with underlying chronic liver disease, including cirrhosis. Organic anion transporter polypeptides (OATPs), encoded by SLCO genes, are one of the most important SLC subfamilies involved in the cellular uptake of drugs and endobiotic. OATP1B1 (SLCO1B1 gene), OATP1B3 (SLCO1B3 gene), and OATP2B1 (SLCO2B1 gene) are hepatic uptake transporters highly expressed in the liver. We aimed to systematically analyze expression levels of SLCO1B1, SLCO1B3, and SLCO2B1 and to investigate their prognostic role in predicting HCC clinical outcomes using open-source databases.

**Methods:**

A comparison of HCC and matched normal tissue gene and protein expression was performed using the TCGA and CPTAC datasets through UALCAN. The correlation between SLCO gene and protein expression with patient survival was evaluated using OncoLnc, KM-Plotter, and OSppc. SLCO genetic alterations in HCC were explored using cBioPortal. A protein-protein interaction map for SLCO1B1, SLCO1B3, and SLCO2B1 was also constructed using STRING.

**Results:**

Gene and protein expression levels of SLCO1B1, SLCO1B3, and SLCO2B1 were significantly downregulated in HCC patients compared to normal counterparts. Clinically, the low gene expression of SLCO1B1, SLCO1B3, and SLCO2B1 was correlated with shorter survival rate in HCC patients. Kaplan-Meier analysis further confirmed that low protein levels of these transporters predicted poor prognosis for HCC patients. Analysis of the TCGA Liver Hepatocellular Carcinoma dataset (*TCGA’s Pan-*Cance*r* Atlas) revealed a low mutation and amplification frequency in HCC for SLCO1B1 (0.57% vs. 0.29%), SLCO1B3 (0.86% vs. 0.29%), and SLCO2B1 (0.57% vs. 0.86%), respectively. Network analysis highlighted non-random interconnectivity among SLCO1B1, SLCO1B3, and SLCO2B1.

**Conclusion:**

SLCO1B1, SLCO1B3, and SLCO2B1 are highly expressed in the *liver* and play key roles in many liver diseases. In *HCC patients*, the downregulation of SLCO1B1, SLCO1B3, and SLCO2B1 expression has been observed. SLCO genes such as SLCO1B1, SLCO1B3, and SLCO2B1 expression levels may also serve as prognostic predictive markers in HCC patients.

## 1 Introduction

Liver cancer remains a significant global health challenge, with its incidence increasing worldwide (Llovet et al., 2016; Villanueva, 2019). Annual cases are projected to exceed one million by 2025 (International Agency for Research on Cancer, 2020). Hepatocellular carcinoma (HCC), the most common form of liver cancer, accounts for approximately 85% of cases (Aly et al., 2020). HCC typically arises in patients with underlying chronic liver disease, particularly cirrhosis, where competing risks of liver failure contribute to low 5-year survival rates of 18%–20% (Davis et al., 2008; Golabi et al., 2017).

The pathophysiology of HCC is complex and occurs through a multistep biological process. The interplay of various factors is at the origin of the early steps of hepatocyte malignant transformation and HCC development. These factors include genetic predisposition, reciprocal interactions between viral and non-viral risk factors, the cellular microenvironment and various immune cells, and the severity of the underlying chronic liver disease (Llovet et al., 2016).

Very few HCC patients are diagnosed during the early stage of the most effective treatment phase (Jessica et al., 2015). Early diagnosis of HCC is crucial, and specific biomarkers for auxiliary examination are tremendously helpful for the prognostic evaluation of HCC patients in clinical practice (Todd et al., 2008; Li et al., 2018). For example, AFP is the most used biomarker in the early detection of HCC and the only biomarker that has been validated for clinical use, but it has limited sensitivity (Parikh et al., 2020). Despite the recent noteworthy improvements in the development of new biomarkers for HCC, it still lacks biomarkers able to predict the prognosis or identify subgroups of patients who would benefit from clinical management (Chen V. L. et al., 2020; Cerrito et al., 2022). Therefore, it is urgent to find other biomarkers to evaluate the prognosis and help clinical management of HCC.

The solute carrier (SLC) superfamily represents the biggest family of transporters with important roles in health and disease. SLC superfamily encompasses many involved in the uptake and distribution of both endogenous compounds and xenobiotics (Hagenbuch and Stieger, 2013). Among these are the organic anion transporting polypeptides (OATPs), sodium-independent plasma membrane transporters encoded by the SCLO genes form the SLC family 21, which transport many structurally diverse amphipathic substrates, bile acids, statins, antihypertensives, antibiotics, antifungals, and chemotherapeutic agents (Hagenbuch and Stieger, 2013; De Bruyn et al., 2011). Organic anion transporting polypeptides 1B1 (OATP1B1, encoded by SLCO1B1), OATP1B3 (encoded by SLCO1B3), and OATP2B1 (encoded by SLCO2B1) are major hepatic uptake transporters (Nies et al., 2013). All three are highly expressed in the liver (Hilgendorf et al., 2007). OATP1B1 is expressed throughout the hepatic lobules, while OATP1B3 expression is concentrated around the central vein (König et al., 2000). Typically, OATP1B1 mRNA levels exceed those of OATP1B3 in the liver (Michalski et al., 2002). Unlike the liver-specific expression of OATP1B1 and OATP1B3, OATP2B1 exhibits a broader tissue expression profile (Savannah et al., 2019). Nevertheless, OATP2B1 mRNA is the most abundant in the liver, and its protein localizes to the basolateral membrane of hepatocytes (Kullak-Ublick et al., 2001). OATPs play important roles in a series of liver diseases, such as hepatitis, liver fibrosis, cirrhosis, and liver cancer (Li et al., 2019). OATP1B1 and OATP1B3 are generally downregulated in hepatic tumors to varying degrees in different studies (Schulte and Ho, 2019). Studies have also shown that decreased expression of OATPs is significantly associated with HCC-related death after relapse (Li et al., 2019). Vasuri et al. correlated the expression of OATP1B1 and OATP1B3 with HCC morphological features and the expression of bile keratin K7 and K19 [associated with a poor prognosis after orthotopic liver transplantation (OLT)] by observing the liver of 69 patients with HCC liver transplantation (OLT) (Vasuri et al., 2011).

In this study, we aimed to systematically analyze expression levels of SLCO1B1, SLCO1B3, and SLCO2B1 and to investigate their prognostic role in predicting HCC clinical outcomes using open-source databases.

## 2 Materials and methods

### 2.1 Gene expression levels of SLCO1B1, SLCO1B3, and SLCO2B1 in normal and cancer tissues

RNA-sequencing data from the GTEx consortium (http://www.gtexportal.org) (Marta et al., 2015) were analyzed to investigate SLCO1B1, SLCO1B3, and SLCO2B1 gene expression levels across human tissues.

All data were browsed and searched by gene symbols. Relationships between SLCO1B1, SLCO1B3, and SLCO2B1 mRNA expression and clinicopathological features, including sex, nodal metastasis status, cancer stage, and tumor grade in HCC patients, were analyzed in the TCGA dataset using UALCAN (http://ualcan.path.uab.edu/index.html) (Chandrashekar et al., 2017). The UALCAN portal features data from the TCGA database, comprising 371 HCC tissue samples and 50 normal liver tissue samples.

### 2.2 Protein expression levels of SLCO1B1, SLCO1B3, and SLCO2B1 in normal and cancer tissues

To further examine protein expression levels of SLCO1B1, SLCO1B3, and SLCO2B1, we also analyzed data from the Clinical Proteomic Tumor Analysis Consortium (CPTAC) dataset using UALCAN (Edwards et al., 2015). The UALCAN portal features data from the CPTCA database, comprising 165 HCC tissue samples and 165 normal liver tissue samples.

### 2.3 Gene expression correlation between SLCO1B1, SLCO1B3, and SLCO2B1 in HCC

Gene expression correlation between SLCO1B1, SLCO1B3, and SLCO2B1 in HCC has been investigated by UALCAN (Chandrashekar et al., 2017). The Pearson correlation coefficient was calculated.

### 2.4 Correlation between SLCO1B1, SLCO1B3, SLCO2B1 expression, and clinical outcomes in HCC

The correlation between SLCO1B1, SLCO1B3, SLCO2B1 gene expression, and the survival rates of HCC patients was assessed using OncoLnc (http://www.oncolnc.org/). This online database provides TCGA survival data related to mRNA, miRNA, and lncRNA expression levels and was used to investigate their prognostic values (Lu et al., 2018). The cut-off value was set as 50%.

We also analyzed the prognostic value of SLCO1B1, SLCO1B3, and SLCO2B1 gene expression in liver cancer patients using Kaplan–Meier plotter (KM plotter) (https://kmplot.com/analysis/), an online database that includes the data from GEO, EGA and TCGA (Lánczky and Győrffy, 2021; Zhang et al., 2023). The KM plotter, handled by a PostgreSQL server, has been widely used to analyze the clinical impact of individual genes on overall survival (OS), relapsed free survival (RFS), progression-free survival (PFS), and disease-specific survival (DSS) of cancer patients. The “Auto select best cutoff” function of KM Plotter was used to compute all possible cut-off values to get the best performing threshold in survival analysis.

Furthermore, using the online consensus survival analysis web server based on Proteome of Pan-cancers (OSppc, https://bioinfo.henu.edu.cn/Protein/OSppc.html), we explored the relationship between protein expression of SLCO1B1, SLCO1B3, SLCO2B1, and the survival rates of HCC patients with the CPTAC dataset (Edwards et al., 2015; Zhang et al., 2023). The cut-off value was set as 50%.

### 2.5 Genetic alteration analysis

Genetic alterations in SLCO1B1, SLCO1B3, and SLCO2B1 were explored within the TCGA Liver Hepatocellular Carcinoma dataset (Pan-Cancer Atlas) using cBioPortal (https://www.cbioportal.org/) (Cerami et al., 2012). The “Oncoprint” and “Cancer Type Summary” tools were employed to investigate mutation frequencies and sites.

### 2.6 Protein-protein interaction

A Protein-protein interaction map for SLCO1B1, SLCO1B3, and SLCO2B1 was constructed using search tool for the retrieval of interacting genes/proteins (STRING v12 https://string-db.org/) (Szklarczyk et al., 2014). Active interaction sources were restricted to “Text mining,” “Experiments” and “Databases.” Only interactions with a high confidence score over 0.7 were mapped to the network. The nodes and edges in the network represent the target genes and their interactions, respectively.

### 2.7 Statistical methods

A Student’s t-test in the UALCAN was performed to *compare* normal and cancer tissues. The correlations between gene expression were evaluated by Pearson’s correlation and statistical significance, and the strength of the correlation was determined using the following guide for the absolute value: 0.00–0.19 “very weak,” 0.20–0.39 “weak,” 0.40–0.59 “moderate,” 0.60–0.79 “strong,” and 0.80–1.0 “very strong.” Survival curves were generated through analysis on OncoLnc online tool, KM plotter, and OSppc. Hazard rate (HR) and P-value or Cox P-value from a log-rank test were displayed through analysis of OncoLnc, KM plotter, and OSppc. A p-value of less than 0.05 was considered statistically significant.

## 3 Results

### 3.1 Expression of SLCO1B1, SLCO1B3, and SLCO2B1 in normal and cancer tissues

**FIGURE 1:**
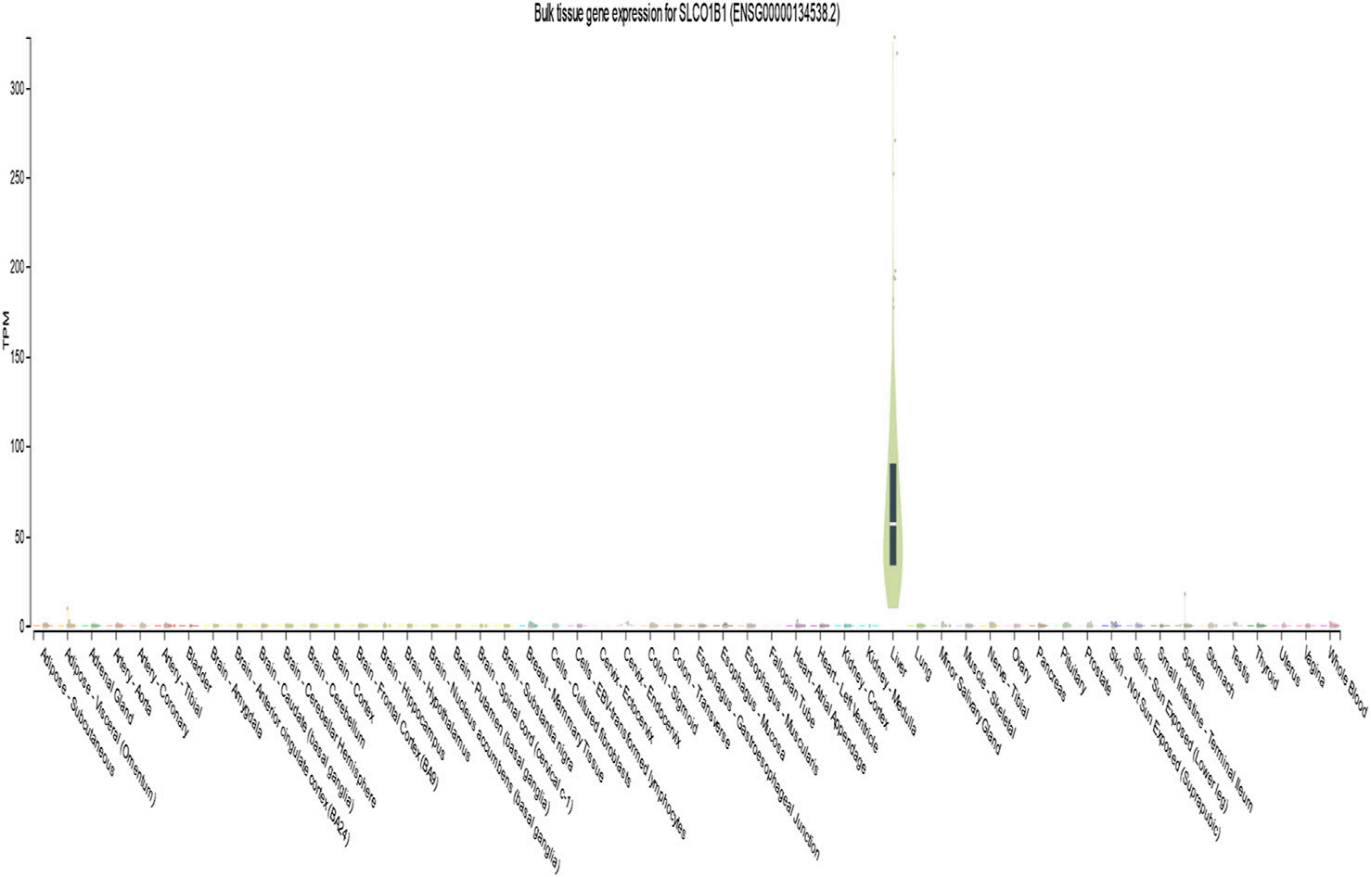
Gene transcript expression levels of SLCO1B1(ENSG00000134538.2) in different normal tissues from GTEx dataset.

Analysis of the GTEx database (version 8) revealed high SLCO1B1 expression in normal liver tissue (median transcripts per million [TPM] = 56.74). In contrast, expression was significantly lower or undetectable in whole blood and other tissues (Figure 1).

**FIGURE 2:**
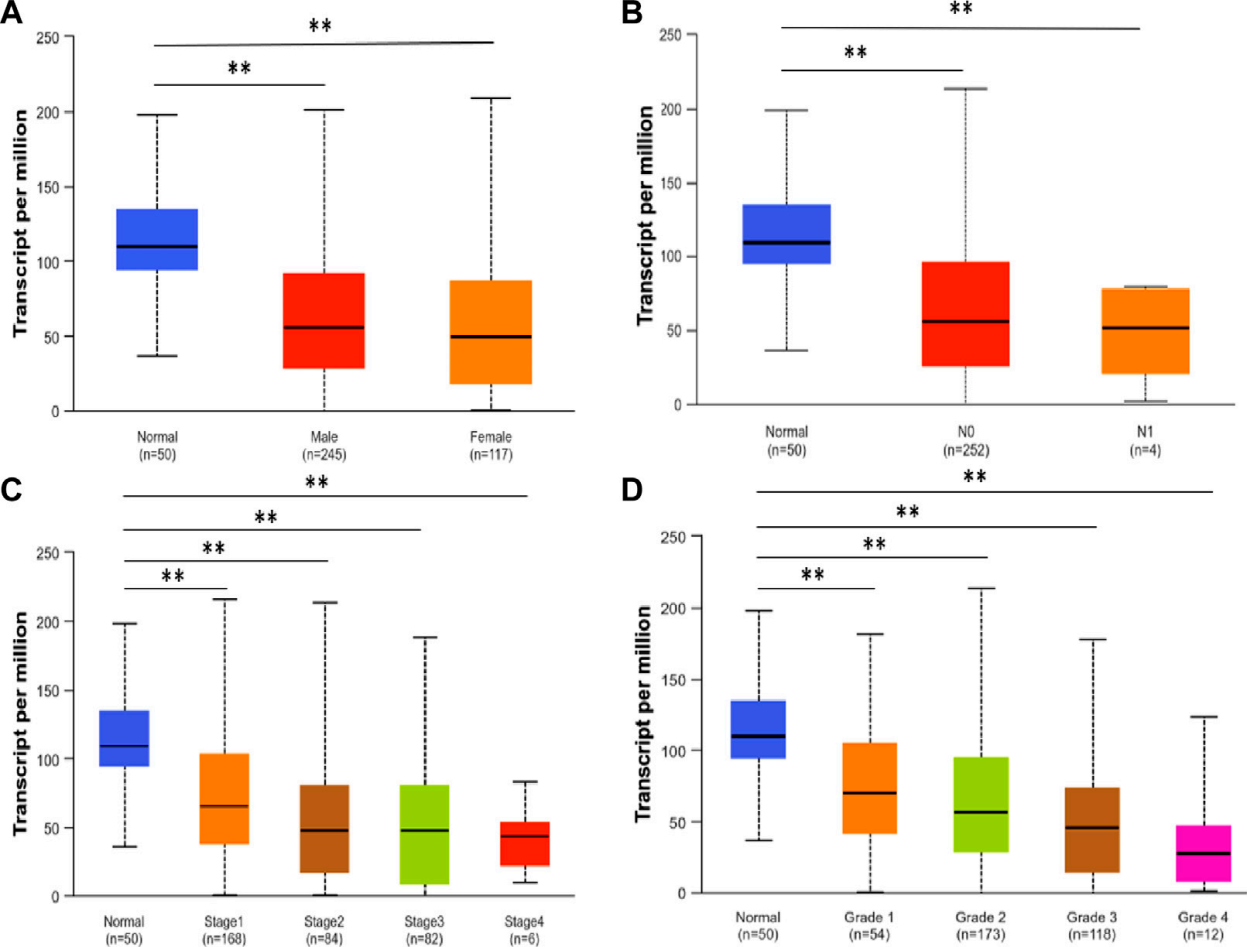
SLCO1B1 gene transcript expression level in normal liver tissues and HCC tissues of different subgroups: **(A)** the relative expression of SLCO1B1 in healthy individuals and male or female patients with HCC; **(B)** the relative expression of SLCO1B1 in healthy individuals and patients with different N stages of HCC **(C)** the relative expression of SLCO1B1 in healthy individuals and patients with different disease stages of HCC **(D)** the relative expression of SLCO1B1 in healthy individuals and patients with different disease grade of HCC. *P < 0.05; **P < 0.01; ns, non-significant.

UALCAN analysis demonstrated significantly reduced SLCO1B1 transcript expression in HCC tissues versus normal liver across all subgroups stratified by gender, nodal metastasis status (N stage), disease stage, and tumor grade (Figures 2A–D).

**FIGURE 3:**
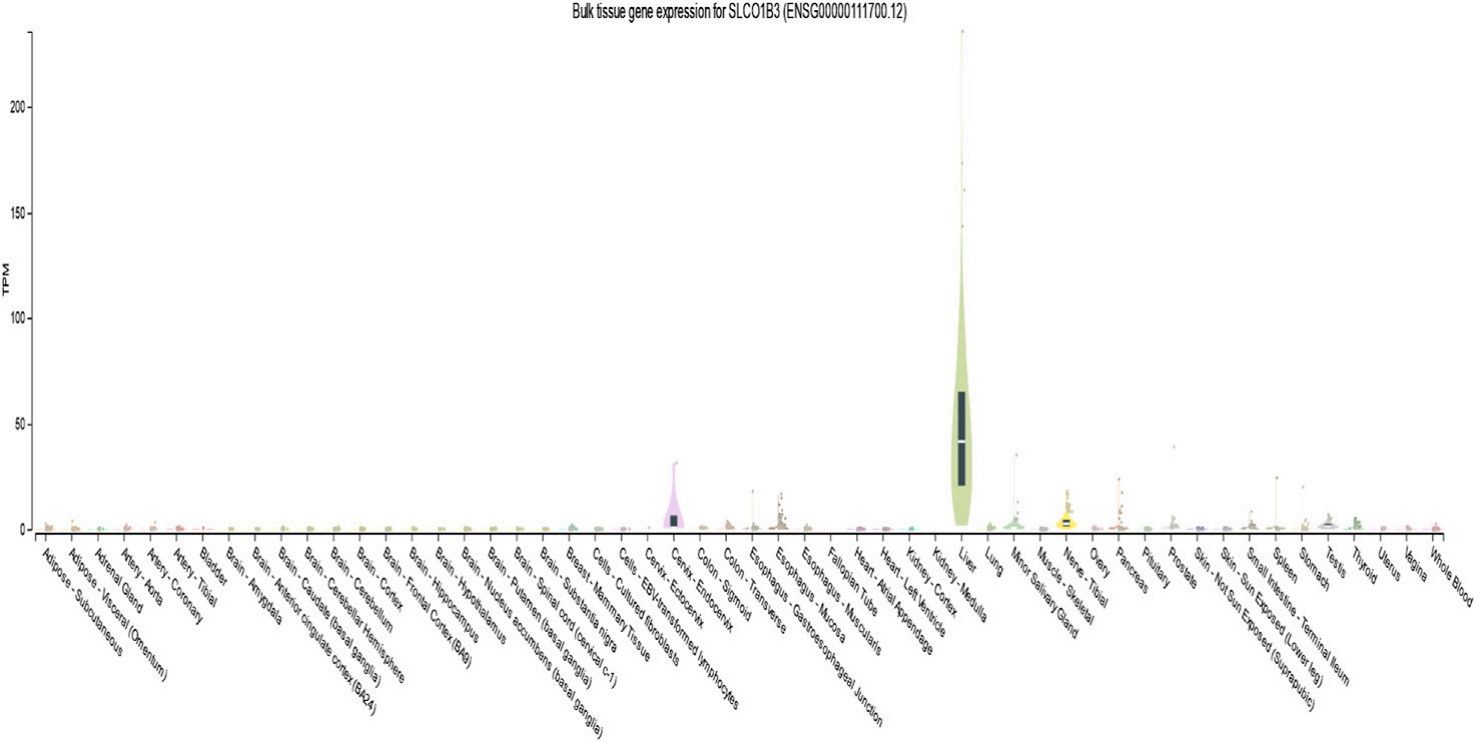
Gene expression for SLCO1B3 (ENSG00000111700.12) among different tissues.

According to the GTEx database (version 8), the expression of SLCO1B3 was high in the liver (median transcripts per million is 41.31). Yet, its expression was very low or no expression in whole blood other tissues (Figure 3).

**FIGURE 4:**
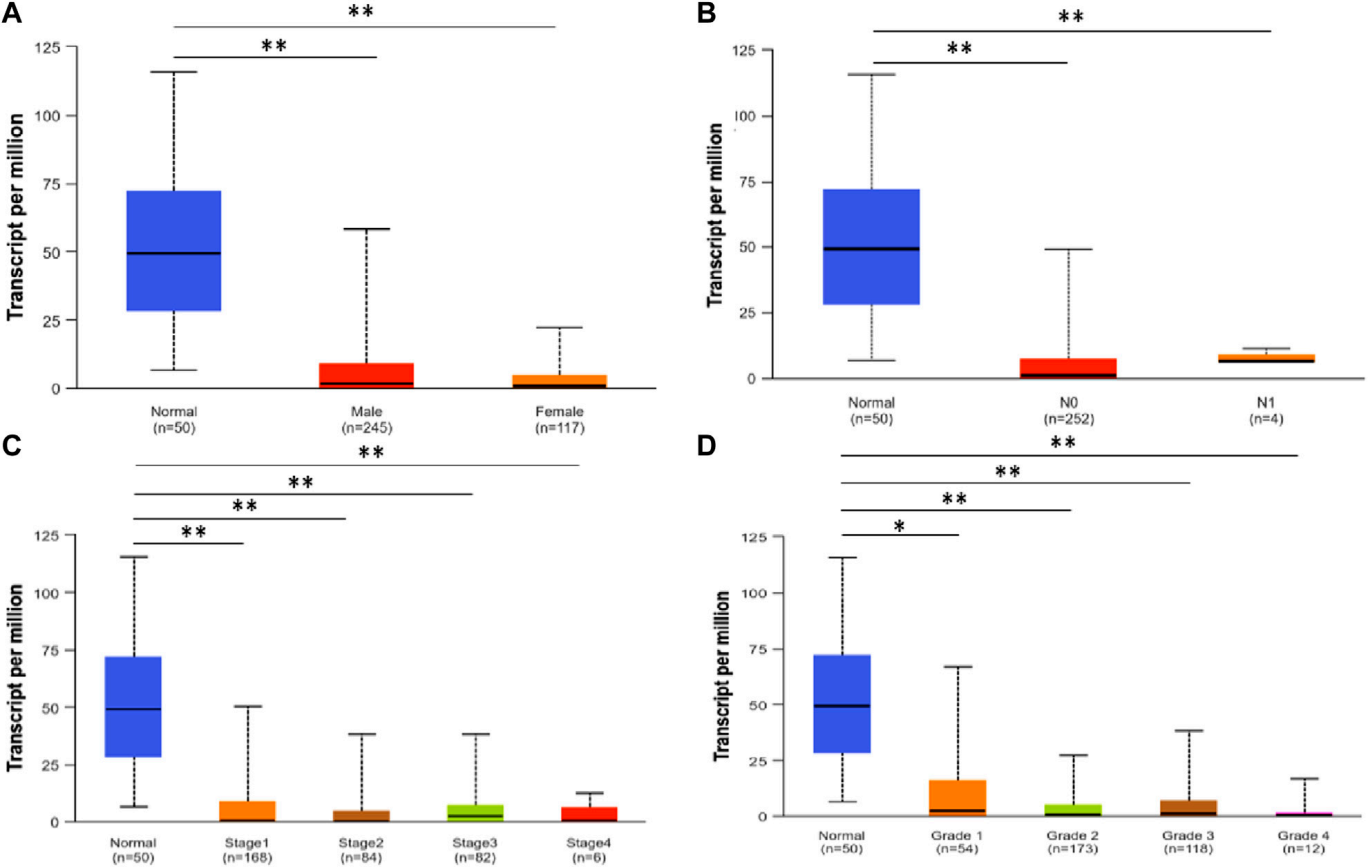
SLCO1B3 transcription in normal liver tissues and HCC tissues of different subgroups: **(A)** the relative expression of SLCO1B3 in healthy individuals and male or female patients with HCC; **(B)** the relative expression of SLCO1B3 in healthy individuals and patients with different N stages of HCC **(C)** the relative expression of SLCO1B3 in healthy individuals and patients with different disease stages of HCC **(D)** the relative expression of SLCO1B3 in healthy individuals and patients with different disease grade of HCC. *P < 0.05; **P < 0.01; ns, non-significant.

Using UALCAN, we analyzed the SLCO1B3 transcript expression level in the normal liver tissues from healthy individuals and cancer liver tissues in HCC patients from different subgroups classified by gender, N stage, disease stage, and disease grade. Consistent with SLCO1B1, SLCO1B3 transcript expression was significantly downregulated in HCC across all clinical subgroups compared to normal liver (Figures 4A–D).

**FIGURE 5:**
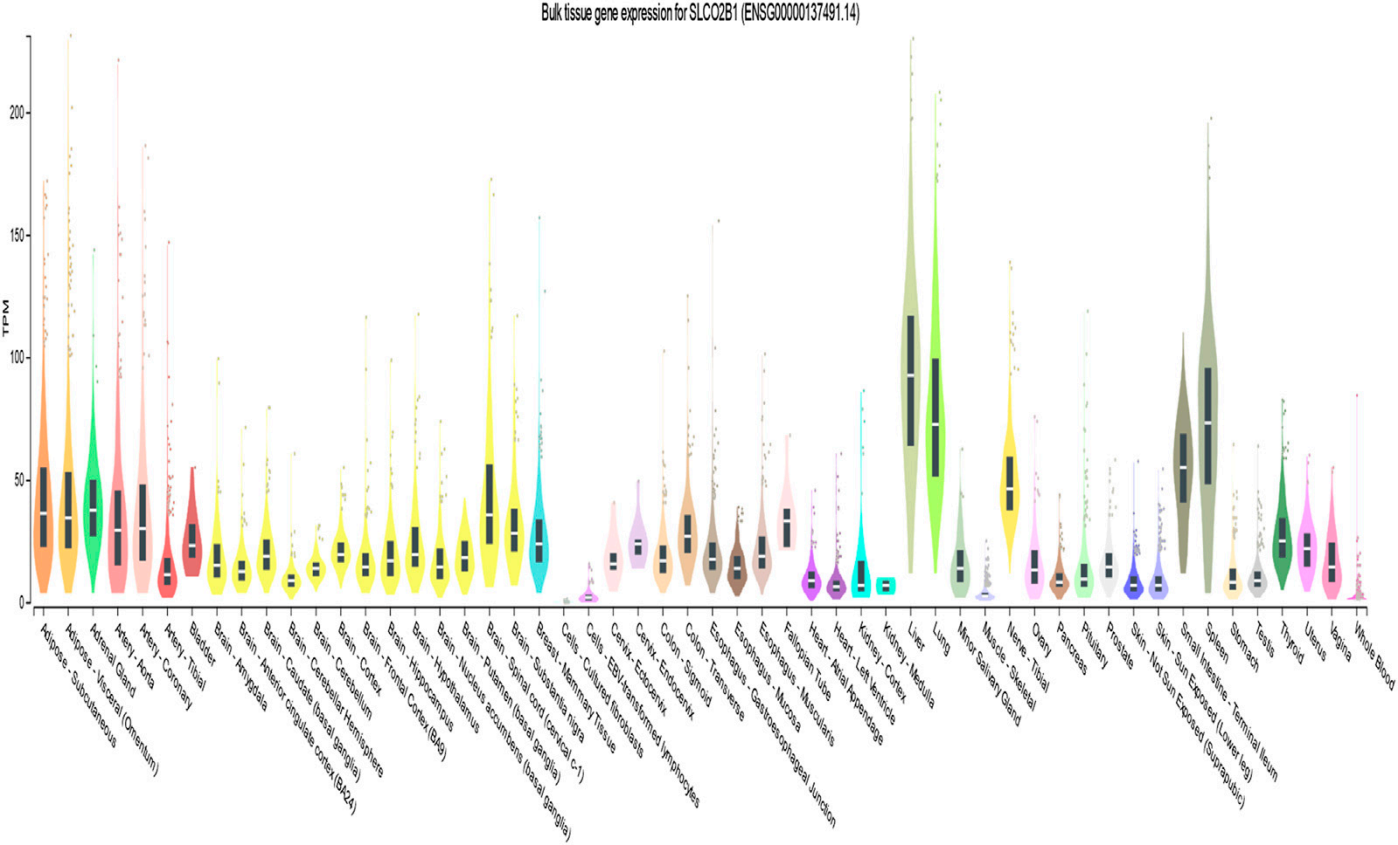
Gene expression for SLCO2B1 (ENSG00000137491.14) among different tissues.

According to the GTEx database, the expression of SLCO2B1 was high in the liver (median transcripts per million (TPM) is 92.50) and has a wide expression profile among different tissues (Figure 5).

**FIGURE 6:**
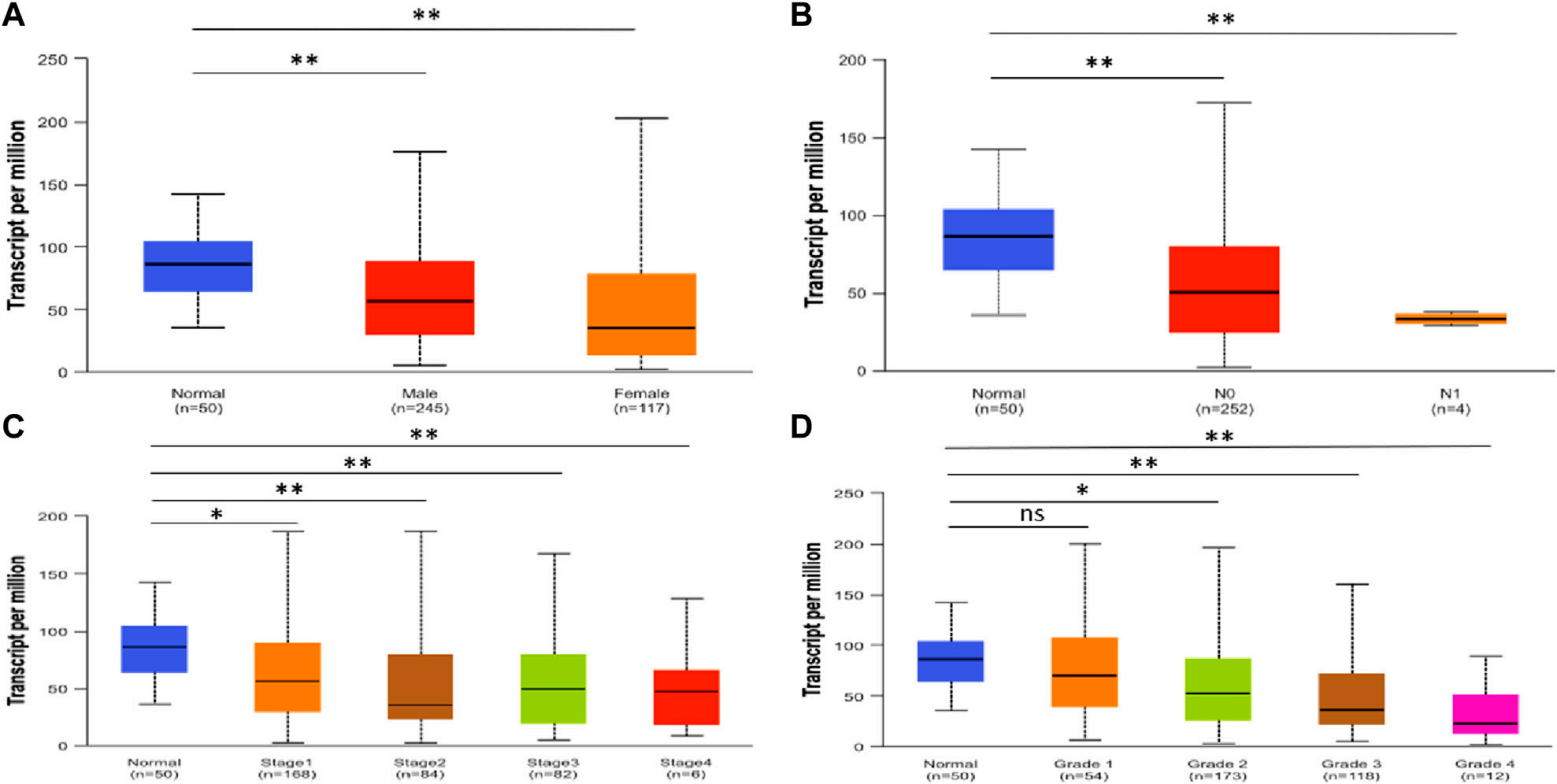
SLCO2B1 transcription in normal liver tissues and HCC tissues of different subgroups: **(A)** the relative expression of SLCO2B1 in healthy individuals and male or female patients with HCC; **(B)** the relative expression of SLCO2B1 in healthy individuals and patients with different N stages of HCC **(C)** the relative expression of SLCO2B1 in healthy individuals and patients with different disease stages of HCC **(D)** the relative expression of SLCO2B1 in healthy individuals and patients with different disease grade of HCC. *P < 0.05; **P < 0.01; ns, non-significant.

Using UALCAN, we analyzed the SLCO2B1 transcript expression level in the normal liver tissues from healthy individuals and cancer liver tissues in HCC patients from different subgroups classified by gender, N stage, disease stage, and disease grade. The results indicated that the SLCO2B1 expression level of HCC groups was significantly lower than those of the normal group, except normal vs. HCC disease grade 1 (Figures 6A–D).

**FIGURE 7:**
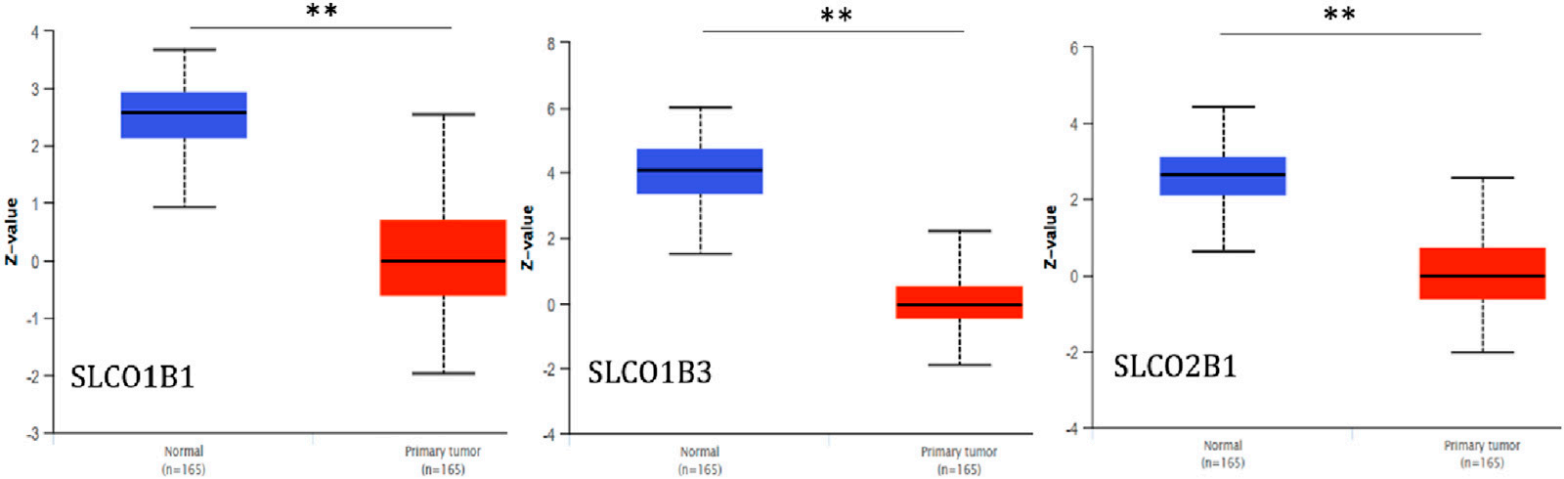
Protein expression levels of SLCO1B1, SLCO1B3, and SLCO2B1 in normal liver tissues and HCC tissues. **P < 0.01.

Using UALCAN, we further examined protein expression levels of SLCO1B1, SLCO1B3, and SLCO2B1 in normal liver tissues from healthy individuals and cancer liver tissues from HCC patients, based on data from the CPTAC dataset. The results indicated that protein expression levels of SLCO1B1, SLCO1B3, and SLCO2B1 were significantly lower in HCC tissues compared to normal liver tissues (Figure 7).

### 3.2 Gene expression correlation between SLCO1B1, SLCO1B3 and SLCO2B1 in HCC

**FIGURE 8:**
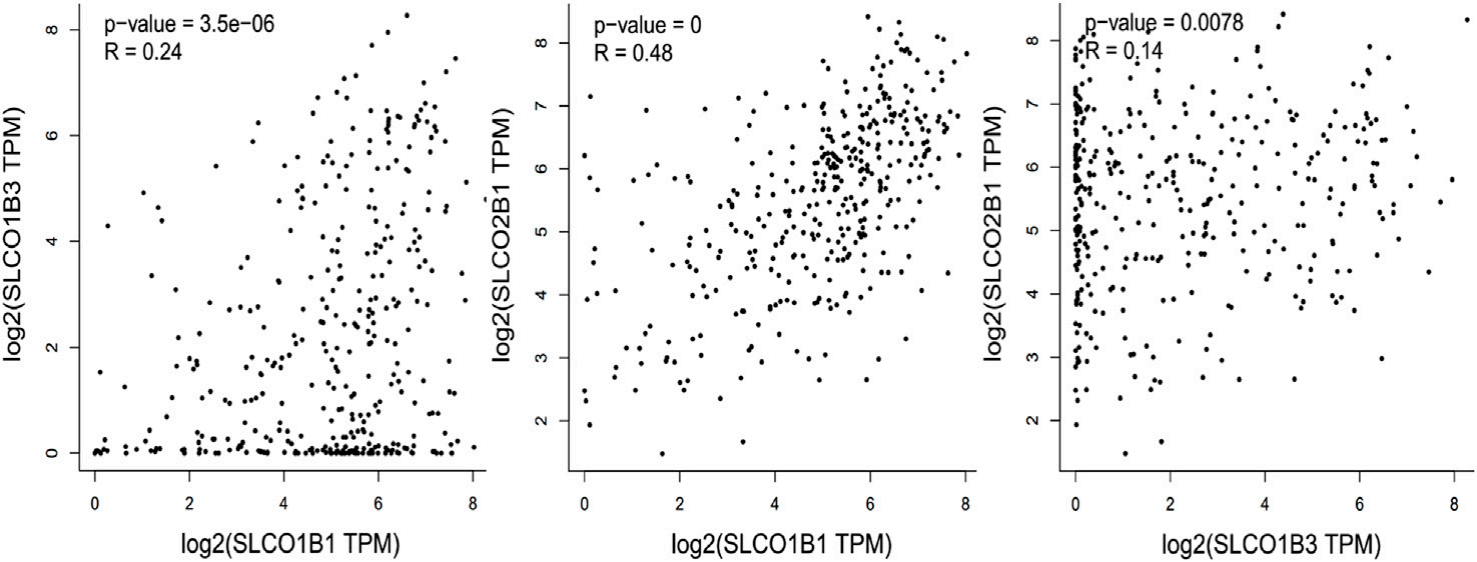
Gene expression correlation between SLCO1B1, SLCO1B3 and SLCO2B1 in HCC.

To investigate the relationship of the expression pattern of the three organic anion transporter polypeptides in HCC, the correlation of the SLCO1B1, SLCO1B3, and SLCO2B1 mRNA expression was analyzed. Using UALCAN, positive correlations of the transcript expression level have been observed among SLCO1B1, SLCO1B3, and SLCO2B1 in HCC, with the moderate positive correlation between SLCO1B1 and SLCO2B1 (Figure 8).

### 3.3 Correlation between SLCO1B1, SLCO1B3, SLCO2B1 expression and clinical outcomes in HCC

**FIGURE 9:**
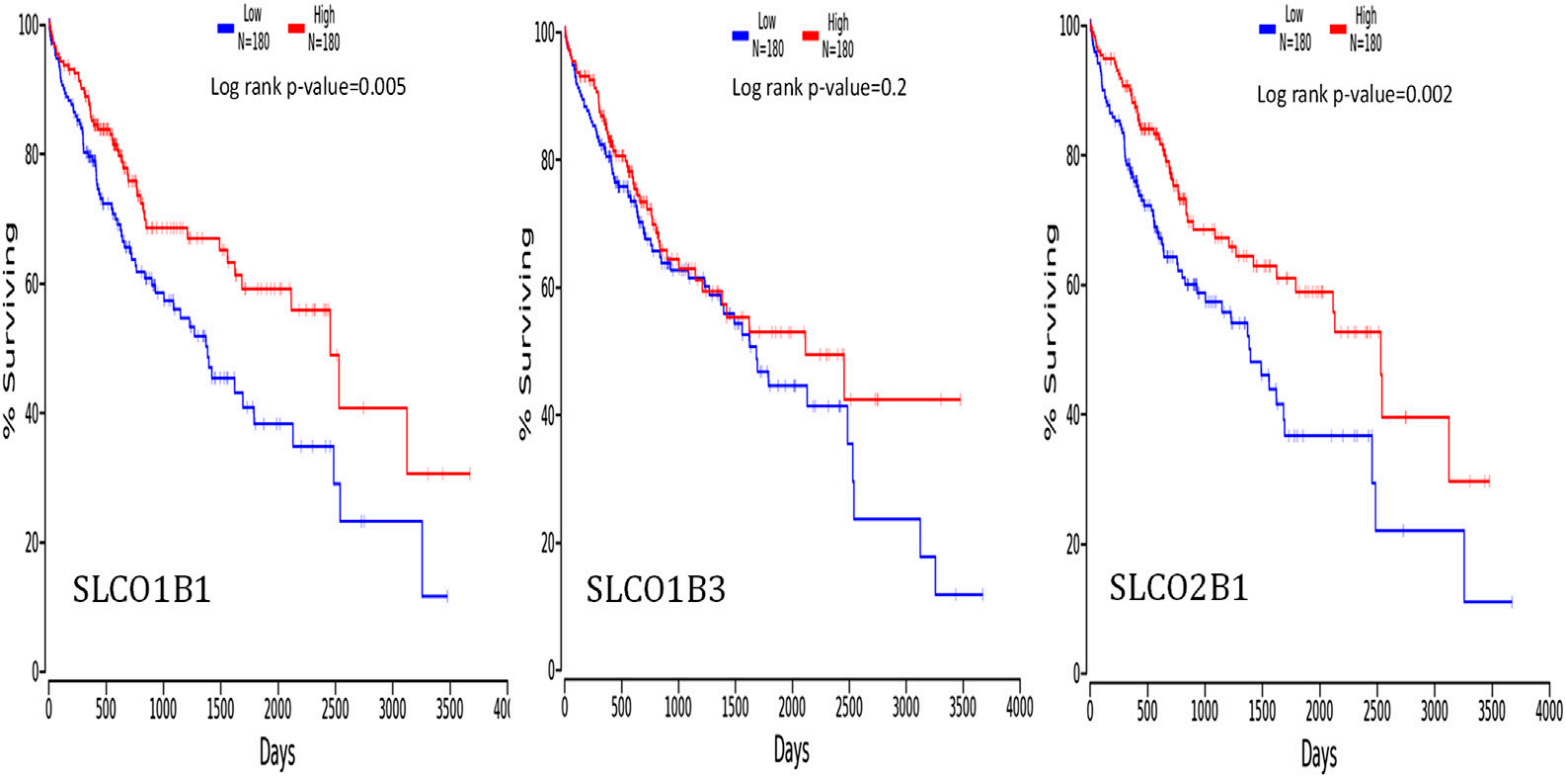
Correlation between the gene expression levels of SLCO1B1, SLCO1B3, and SLCO2B1, and survival outcomes using the OncoLnc online tool. The cut-off value was 50%.

To investigate whether the overall survival outcome of HCC patients is related to the gene expression levels of SLCO1B1, SLCO1B3, and SLCO2B1, we compared the overall survival rate of patients with a high gene expression level of SLCO1B1, SLCO1B3, and SLCO2B1 to those with a low gene expression level of SLCO1B1, SLCO1B3, and SLCO2B1 using the OncoLnc online tool (Figure 9). The results showed the relationship with survival after regression analysis and multivariate analysis performed controlling for other relevant variables. Notably, the low gene expression of SLCO1B1 and SLCO2B1 are correlated significantly with a shorter overall survival in HCC patients, with a log-rank p-value of 0.005 and 0.002, respectively. S*urvival* analysis indicated a *trend in* shorter overall *survival* with low gene expression of SLCO1B3 in HCC patients; however, there was no significant correlation between SLCO1B3 gene expression and the survival rates (p = 0.2).

**FIGURE 10:**
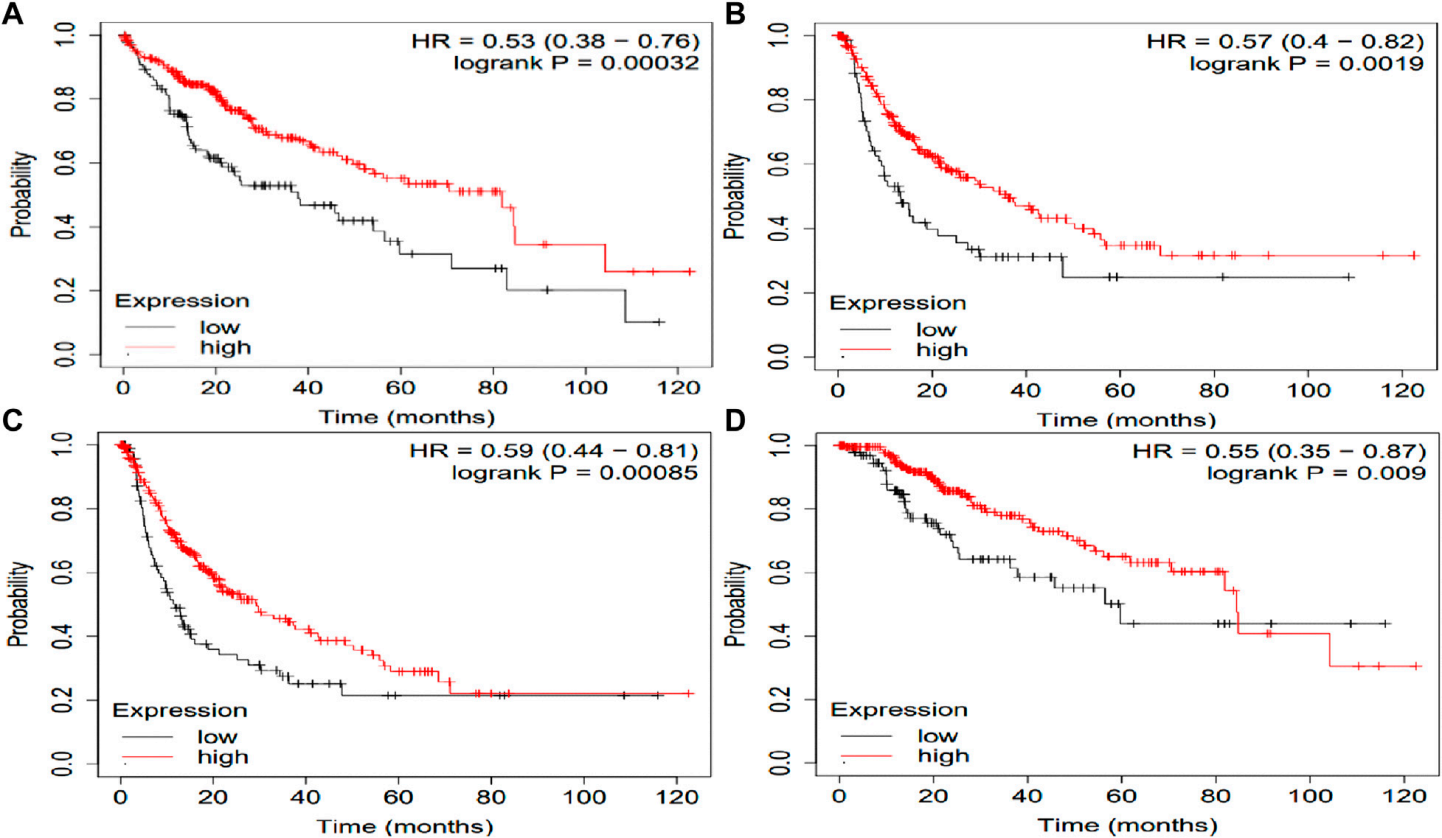
Kaplan–Meier curves of clinical outcomes of HCC comparing the high and low gene expression of SLCO1B1 in the KM plotter. **(A)** OS survival curves (*n* = 364); **(B)** RFS survival curves (*n* = 316); **(C)** PFS survival curves (*n* = 370); **(D)** DSS survival curves (*n* = 362). Auto select best cutoff function of KM Plotter was used.

**FIGURE 11:**
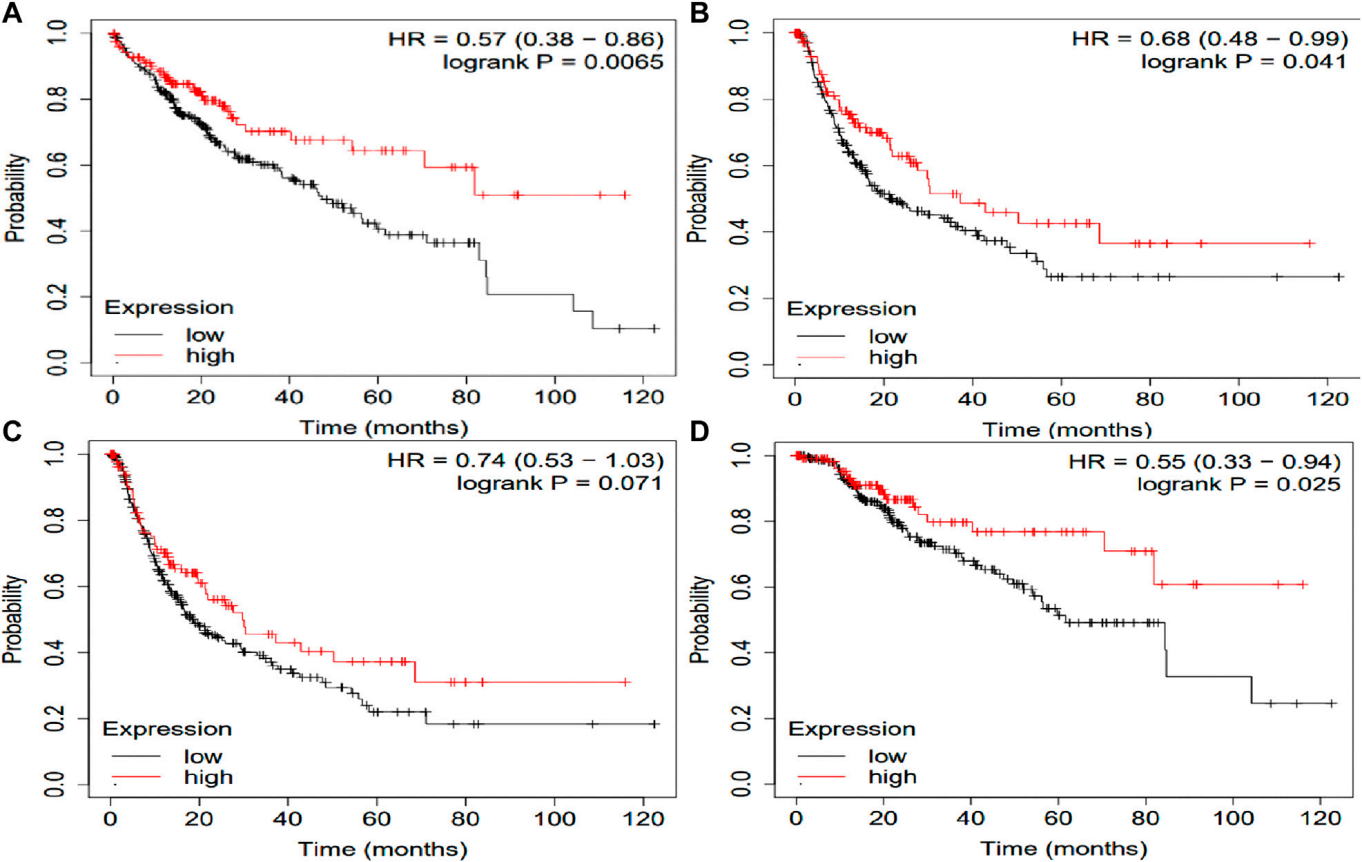
Kaplan–Meier curves comparing the high and low gene expression of SLCO1B3 in liver cancer in the KM plotter. **(A)** OS survival curves (*n* = 364); **(B)** RFS survival curves (*n* = 316); **(C)** PFS survival curves (*n* = 370); **(D)** DSS survival curves (*n* = 362). Auto select best cutoff function of KM Plotter was used.

**FIGURE 12:**
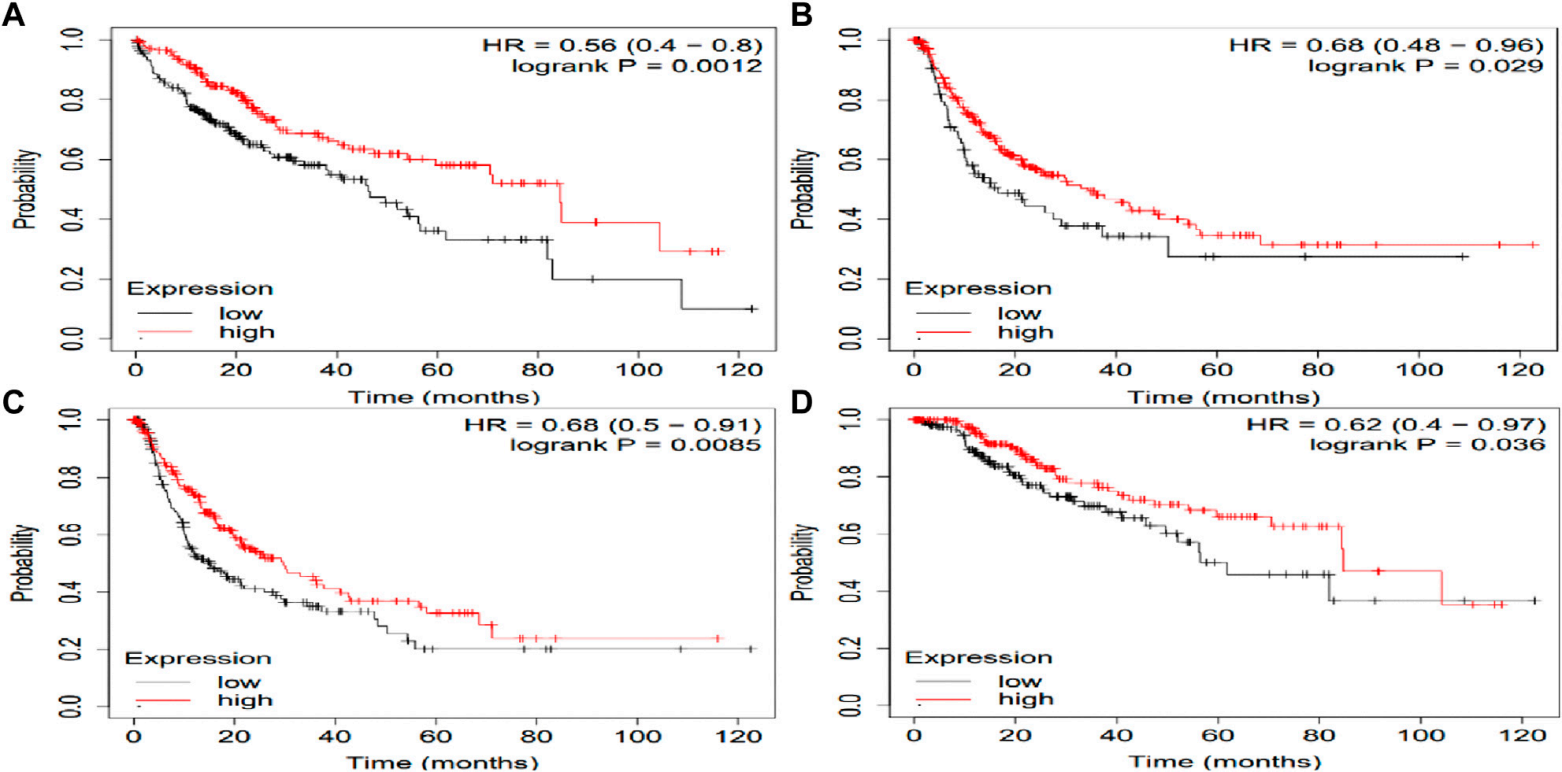
Kaplan–Meier curves comparing the high and low gene expression of SLCO2B1 in liver cancer in the KM plotter. **(A)** OS survival curves (*n* = 364); **(B)** RFS survival curves (*n* = 316); **(C)** PFS survival curves (*n* = 370); **(D)** DSS survival curves (*n* = 362). Auto select best cutoff function of KM Plotter was used.

The potential prognosis value of SLCO1B1, SLCO1B3 and SLCO2B1 in predicting clinical outcomes of HCC patients was also investigated through KM plotter (Figures 10–12). Through KM plotter analysis in HCC patients, SLCO1B1 and SLCO2B1 gene expressions were significantly correlated with OS, RFS, PPS and DSS. SLCO1B1 gene expression was significantly correlated with OS, RFS and DSS, while there was no statistical significance in PFS. In general, low gene expression levels of SLCO1B1, SLCO1B3 and SLCO2B1 predict poor prognosis in HCC patients.

**FIGURE 13:**
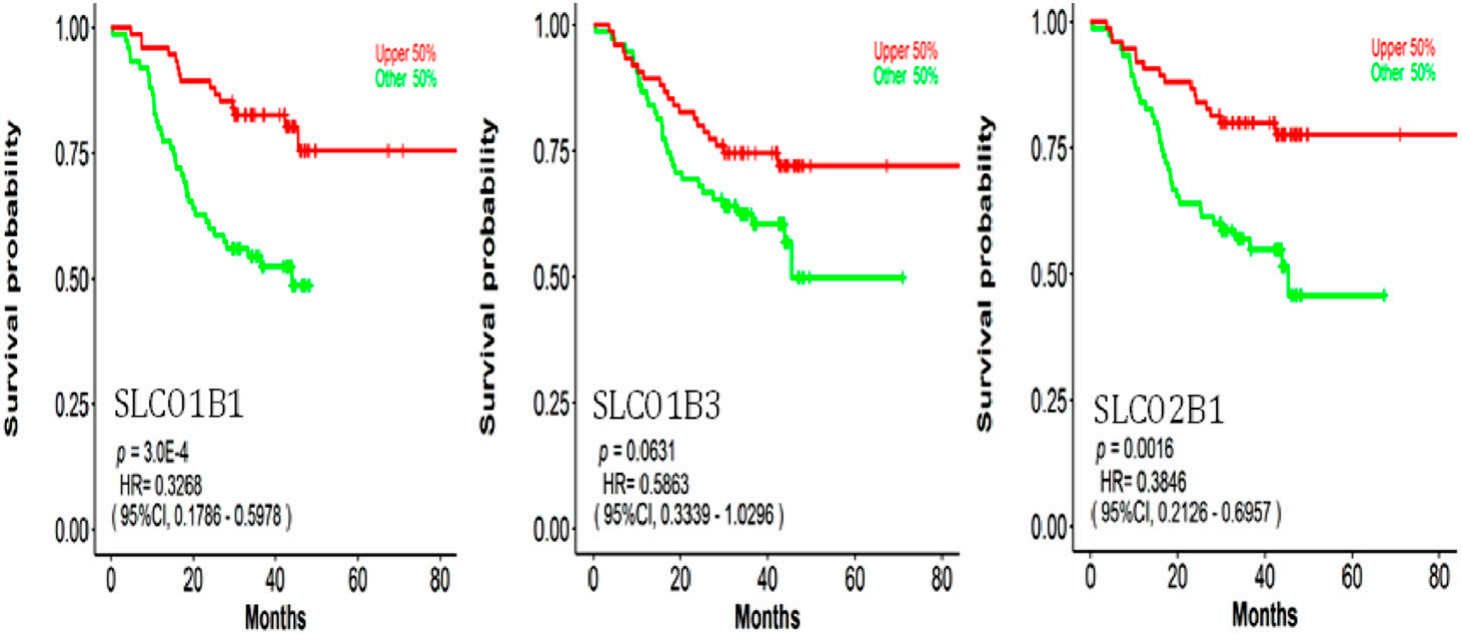
Correlation between SLCO1B1, SLCO1B3, and SLCO2B1 protein expression levels and survival outcomes using the OSppc online tool. The cut-off value was 50%.

We further explored the relationship between protein expressions of SLCO1B1, SLCO1B3, SLCO2B1, and the survival rates of HCC patients with the CPTAC dataset (Figure 13).

Low protein expressions of SLCO1B1, and SLCO2B1 were significantly correlated with a shorter overall survival in HCC patients, with a log-rank p-value of 0.0003 and 0.0016, respectively. Kaplan–Meier *survival* analysis indicated a *trend in* shorter overall *survival* with low protein expression of SLCO1B3 in HCC patients, with a log-rank p-value of 0.0631.

### 3.4 Mutation and amplification of SLCO1B1, SLCO1B3, and SLCO2B1 in HCC

**FIGURE 14:**
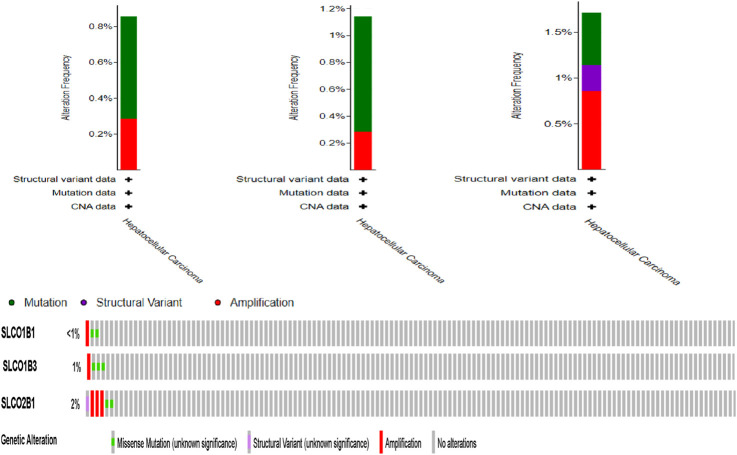
Frequency of SLCO1B1, SLCO1B3, and SLCO2B1 mutation and amplification frequency in HCC patients (cBioportal).

cBioPortal analysis of TCGA data revealed low mutation/amplification frequencies: SLCO1B1 (0.57% mutation vs. 0.29% amplification), SLCO1B3 (0.86% vs. 0.29%), and SLCO2B1 (0.57% vs. 0.86%) (Figure 14).

### 3.5 Network analysis highlights non-random interconnectivity among SLCO1B1, SLCO1B3 and SLCO2B1

**FIGURE 15:**
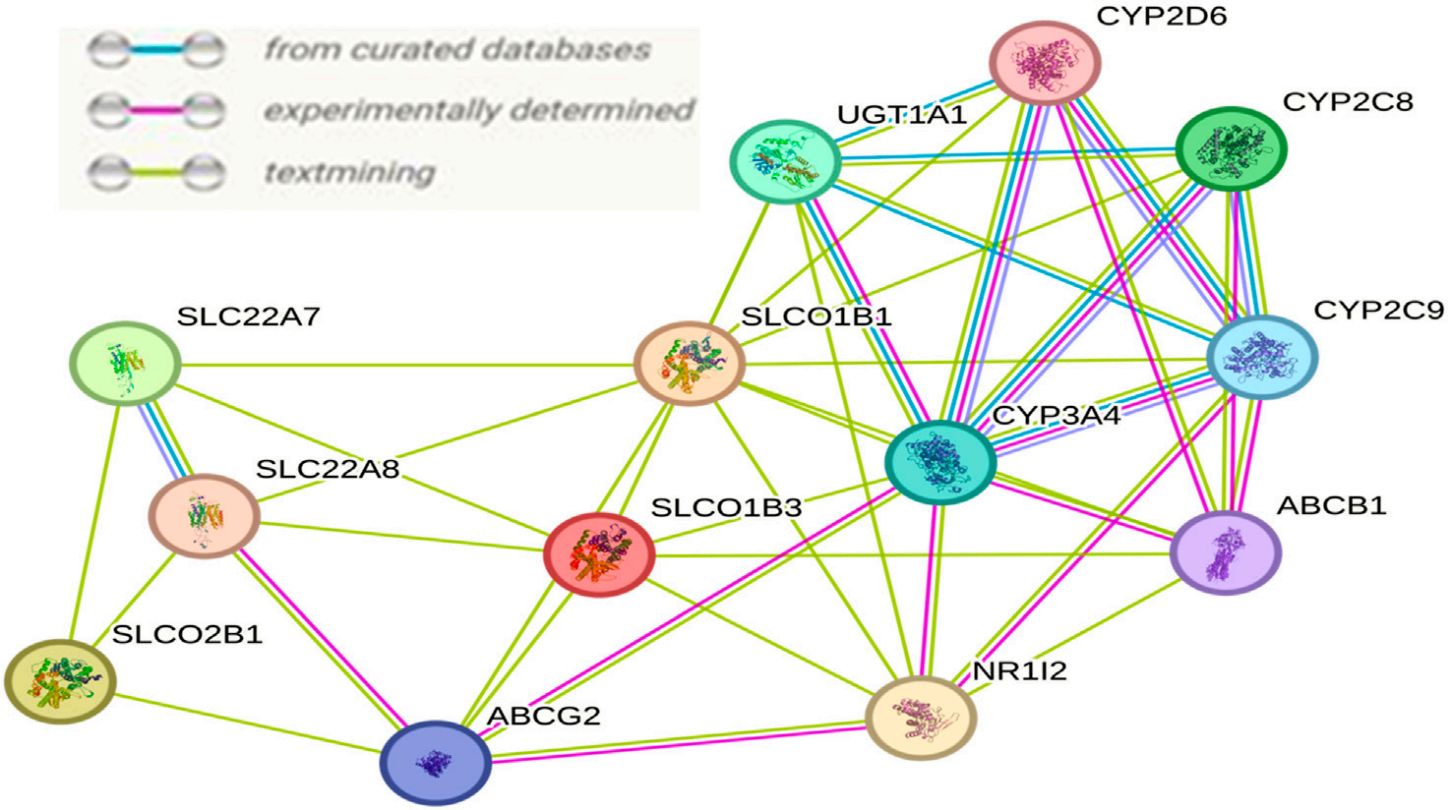
Interaction network resulting among SLCO1B1, SLCO1B3, and SLCO2B1 generated by the STRING.

Interactions among SLCO1B1, SLCO1B3, and SLCO2B1 with other proteins using STRING describe various interacting partners. SLCO1B1, SLCO1B3 and SLCO2B1 were input as the “seed” proteins to construct the PPI network (Figure 15). To identify the most significant interactions and achieve a meaningful size for network analysis, ten additional interactors were allowed in the network. The network enrichment p-value was <4.85e-13, meaning that this connected network has significantly more interactions than expected at random. Such enrichment also indicates that SLCO1B1, SLCO1B3, and SLCO2B1 are, at least partially, biologically connected.

## 4 Discussion

Hepatocellular carcinoma, one of the most prevalent cancers globally, remains a significant public health burden (Llovet et al., 2016; Villanueva, 2019). Established major risk factors include chronic cirrhosis, viral hepatitis, nonalcoholic fatty liver disease, alcohol use, and genetic disorders (Davis et al., 2008; Golabi et al., 2017). Diagnosing HCC, particularly in its early stages, remains challenging (Jessica et al., 2015). Early diagnosis is crucial, and specific biomarkers for auxiliary examination are highly valuable for prognostic evaluation in clinical practice (Todd et al., 2008; Li et al., 2018).

Sodium-independent plasma membrane transporters encoded by the SCLO genes play important roles in the uptake and distribution of both endogenous compounds and xenobiotics (Hagenbuch and Stieger, 2013). OATPs play important roles in a series of liver diseases, such as hepatitis, liver fibrosis, and cirrhosis (Li et al., 2019). The hepatitis-fibrosis-cirrhosis progression eventually leads to liver cancer.

OATP1B1 (encoded by the SLCO1B1 gene) and OATP1B3 (encoded by the SLCO1B3 gene) are expressed mainly in liver cells. OATP2B1 (encoded by the SLCO2B1 gene) is also highly expressed in the liver. In contrast to the liver-specific expression of OATP1B1 and 1B3, OATP2B1 has a wide expression profile. Using UALCAN, gene transcript expression levels of SLCO1B1, SLCO1B3, and SLCO2B1 were shown to be significantly downregulated in the clinic-pathological characteristics (gender, nodal metastasis status, cancer stages and tumor grade) in HCC patients compared to normal counterparts. In addition, protein expression levels of SLCO1B1, SLCO1B3, and SLCO2B1 were also significantly lower in HCC tissues compared to normal liver tissues. This is consistent with earlier reports from other researchers that downregulation in tumor tissue OATP1B1, OATP1B3, and OTTP2B1 expression is markedly reduced in HCC tumor tissues compared to adjacent non-tumorous liver, as shown by immunohistochemistry and mRNA/protein analyses (Wlcek et al., 2011; Billington et al., 2018; Thakkar et al., 2017; Le Vee et al., 2011; Chen Shihan et al., 2020). This finding suggests that SLCO1B1, SLCO1b3, and SLCO2B1 expression levels may be used for assessing the risk of HCC development. Further empirical validation through experimental approaches (e.g., immunohistochemistry or functional assays) is crucial to reinforce the biological relevance of the results.

Using UALCAN, positive correlations were observed among the gene expression of SLCO1B1, SLCO1B3, and SLCO2B1 in HCC, with the strongest positive correlation between SLCO1B1 and SLCO2B1. Analysis of the TCGA Liver Hepatocellular Carcinoma dataset (TCGA’s Pan-Cancer Atlas) revealed a low mutation and amplification frequency in HCC for SLCO1B1 (0.57% vs. 0.29%), SLCO1B3 (0.86% vs. 0.29%) and SLCO2B1 (0.57% vs. 0.86%).

Multivariate Cox regression analysis performed using OncoLnc demonstrated that low SLCO1B1 and SLCO2B1 gene expression levels significantly correlated with shorter overall survival in HCC patients (p = 0.005 and p = 0.002, respectively). Although low SLCO1B3 expression showed a trend towards shorter overall survival, this correlation was not statistically significant (p = 0.2). The KM plotter analysis indicated the same trend, low expression of these transporters associated with shorter OS, RFS, PFS and DSS. Additionally, survival analysis through OSppc further confirmed that low protein levels of these transporters predicted poor prognosis for HCC patients.

Our findings align with previous reports. For instance, Shihan Chen et al. indicated that low OATP1B3 levels are independently associated with larger tumor size, higher recurrence, poor differentiation, advanced tumor node metastasis stage, and significantly shorter overall and disease-free survival (Chen Shihan et al., 2020). They further demonstrated that OATP1B3 overexpression in HCC cells promotes apoptosis and suppresses proliferation, while low OATP1B3 expression predicts a poor prognosis (Chen Shihan et al., 2020) Downregulation of OATP1B1, OATP1B3, and OATP2B1 in HCC may follow a common mechanism, potentially influenced by the increased expression of proinflammatory cytokines, such as TNF-α, IL-6, and IFN-γ. These changes are observed at mRNA levels, but also, at protein and activity levels (Fardel and Le Vée, 2009; Korobkova, 2015). Hao Tianran et al. also reported that exposure to a cytokine cocktail containing IL-1, TNF-α, and IFN-γ significantly downregulates mRNA and activity of OATP1B1, OATP1B3, and OATP2B1 in hepatocytes (Tianran et al., 2024). Additionally, elevated levels of IL-6, and TNF-α, are observed in HCC patients and are associated with inflammation-driven carcinogenesis, activation of oncogenic pathways (e.g., STAT3), and poor prognosis (Kao et al., 2015). These findings highlight the mechanistic connections between these cytokines and OATPs regulation in the context of HCC, suggesting that SLCO1B1, SLCO1B3, and SLCO2B1 may be prognostic factors for liver cancer patients.

SLCO1B1, SLCO1B3, and SLCO2B1 also interact with various cytochrome P450 (CYP) enzymes, including CYP3A4, CYP2D6, CYP2C8, and CYP2C9. Cytochrome P450s, localized to mitochondrial membranes or the endoplasmic reticulum, are crucial for metabolizing a wide range of endogenous and exogenous compounds (Sheweita, 2000). Various cytotoxic drugs can be activated or inactivated by drug-metabolizing enzymes in tumor tissues, thereby affecting the susceptibility of both the host and the tumor to their effects (Yan et al., 2015). The activities and protein amount of major CYP enzymes were found to be significantly decreased in HCC tumors (Oyama et al., 2004). It has been suggested that the local expression of CYPs in tumors is essential in the management of cancer since these functionally associated enzymes might contribute both in the development of HCC and in determining the anticancer drug sensitivity (Michael and Doherty, 2007; Ren et al., 2018). OATPs play a vital role in facilitating the cellular uptake of drugs and endogenous compounds, interact with various CYP enzymes which are key players in drug metabolism. Decreased expression of OATP1B1 and OATP1B3 is associated with potential resistance to anticancer drugs that rely on these transporters for cellular uptake, contributing to poor prognosis in HCC patients (Zhu et al., 2024; Wen and Zhao, 2021). Maitane Asensio et al., reported relevance of the OATP1B3 in the personalized pharmacological treatment of hepatocellular carcinoma, and recommended Lt-OATP1B3 expression should be screened prior to deciding the use of anticancer drugs substrates of this carrier in the personalized treatment of HCC (Sanchez-Vicente et al., 2023). Understanding the relationship between OATPs and drug sensitivity is an important area of study. Investigating these connections through multi-omics integration analysis can provide deeper insights into the biological functions of OATPs in HCC, potentially uncovering their impact on therapeutic strategies.

## 5 Conclusion

SLCO1B1, SLCO1B3, and SLCO2B1 are highly expressed in the liver and play key roles in various liver diseases. Our study confirms the downregulation of SLCO1B1, SLCO1B3, and SLCO2B1 expression in HCC patients. Furthermore, the expression levels of these SLCO genes may serve as prognostic markers in HCC patients.

## Data Availability

Publicly available datasets were analyzed in this study. This data can be found here: http://www.gtexportal.org (GTEx consortium) http://ualcan.path.uab.edu/index.html; http://www.oncolnc.org/ (TCGA dataset in UALCAN and OncoLnc). https://kmplot.com/analysis/; https://bioinfo.henu.edu.cn/Protein/OSppc.htm; https://www.cbioportal.org/ https://string-db.org/.

## References

[B1] Aly A. Ronnebaum S. Patel D. Doleh Y. Benavente F. (2020). Epidemiologic, humanistic and economic burden of hepatocellular carcinoma in the USA: asystematic literature review. Hepat. Oncol. 7 (3), HEP27. 10.2217/hep-2020-0024 32774837 PMC7399607

[B2] Billington S. Ray A. S. Salphati L. Xiao G. Chu X. Humphreys W. G. (2018). Transporter expression in noncancerous and cancerous liver tissue from donors with hepatocellular carcinoma and chronic hepatitis C infection quantified by LC-MS/MS proteomics. Drug Metab. Dispos. 46 (2), 189–196. 10.1124/dmd.117.077289 29138286 PMC5776333

[B3] Cerami E. Gao J. Dogrusoz U. Gross B. E. Sumer S. O. Aksoy B. A. (2012). The cBio cancer genomics portal: an open platform for exploring multidimensional cancer genomics data. Cancer Discov. 2, 401–404. 10.1158/2159-8290.CD-12-0095 22588877 PMC3956037

[B4] Cerrito L. Ainora M. E. Mosoni C. Borriello R. Gasbarrini A. Zocco M. A. (2022). Prognostic role of molecular and imaging biomarkers for predicting advanced hepatocellular carcinoma treatment efficacy. Cancers 14 (19), 4647. 10.3390/cancers14194647 36230569 PMC9564154

[B5] Chandrashekar D. S. Bashel B. Balasubramanya S. A. H. Creighton C. J. Ponce-Rodriguez I. Chakravarthi B. V. S. K. (2017). UALCAN: a portal for facilitating tumor subgroup gene expression and survival analyses. Neoplasia 19 (8), 649–658. 10.1016/j.neo.2017.05.002 28732212 PMC5516091

[B6] Chen S. Li K. Jiang J. Wang X. Chai Y. Zhang C. (2020b). Low expression of organic anion-transporting polypeptide 1B3 predicts a poor prognosis in hepatocellular carcinoma. J. Surg. Oncol. 18, 127. 10.1186/s12957-020-01891-y PMC7293789 32534581

[B7] Chen V. L. Xu D. Wicha M. S. Lok A. S. Parikh N. D. (2020a). Utility of liquid biopsy analysis in detection of hepatocellular carcinoma, determination of prognosis, and disease monitoring: a systematic review. Clin. Gastroenterol. Hepatol. 18 (13), 2879–2902. 10.1016/j.cgh.2020.04.019 32289533 PMC7554087

[B8] Davis G. L. Dempster J. Meler J. D. Orr D. W. Walberg M. W. Brown B. (2008). Hepatocellular carcinoma: management of an increasingly common problem. Proc. Bayl Univ. Med. Cent. 21 (3), 266–280. 10.1080/08998280.2008.11928410 18628926 PMC2446418

[B9] De Bruyn T. Ye Z. W. Peeters A. Sahi J. Baes M. Augustijns P. F. (2011). Determination of OATP-NTCP- and OCT-mediated substrate uptake activities in individual and pooled batches of cryopreserved human hepatocytes. Eur. J. Pharm. Sci. 43, 297–307. 10.1016/j.ejps.2011.05.002 21605667

[B10] Edwards N. J. Oberti M. Thangudu R. R. Cai S. McGarvey P. B. Jacob S. (2015). The CPTAC data portal: a resource for cancer proteomics research. J. Proteome Res. 14, 2707–2713. 10.1021/pr501254j 25873244

[B11] Fardel O. Le Vée M. (2009). Regulation of human hepatic drug transporter expression by pro-inflammatory cytokines. Expert Opin. Drug Metab. Toxicol. 5, 1469–1481. 10.1517/17425250903304056 19785515

[B12] Golabi P. Fazel S. Otgonsuren M. Sayiner M. Locklear C. T. Younossi Z. M. (2017). Mortality assessment of patients with hepatocellular carcinoma according to underlying disease and treatment modalities. Medicine 96, e5904. 10.1097/MD.0000000000005904 28248853 PMC5340426

[B13] Hagenbuch B. Stieger B. (2013). The SLCO (former SLC21) superfamily of transporters. Mol. Asp. Med. 34, 396–412. 10.1016/j.mam.2012.10.009 PMC3602805 23506880

[B14] Hilgendorf C. Ahlin G. Seithel A. Artursson P. Ungell A. L. Karlsson J. (2007). Expression of thirty-six drug transporter genes in human intestine, liver, kidney, and organotypic cell lines. Drug Metab. Dispos. 35, 1333–1340. 10.1124/dmd.107.014902 17496207

[B15] International Agency for Research on Cancer (2020). GLOBOCAN 2018. IARC. Available online at: https://gco.iarc.fr/today/onlineanalysismap?v=2020&mode=population&mode_population=continents&population=900&populations=900&key=asr&sex=0&cancer=11&type=0&statistic=5&prevalence=0&population_groupearth&color_palette=default&map_scale=quantile&map_nb_colors=5&continent=0&rotate=%255B10%252C0%255D.

[B16] Jessica Z.-R. Augusto V. Jean-Charles N. Llovet J. M. (2015). Genetic landscape and biomarkers of hepatocellular carcinoma. Gastroenterology 149, 1226–1239.e4. 10.1053/j.gastro.2015.05.061 26099527

[B17] Kao J.-T. Feng C.-L. Yu C.-J. Tsai S.-M. Hsu P.-N. Chen Y.-L. (2015). IL-6, through p-STAT3 rather than p-STAT1, activates hepatocarcinogenesis and affects survival of hepatocellular carcinoma patients: a cohort study. BMC Gastroenterol. 15, 50. 10.1186/s12876-015-0283-5 25908103 PMC4424593

[B18] König J. Cui Y. Nies A. T. Keppler D. (2000). Localization and genomic organization of a new hepatocellular organic anion transporting polypeptide. J. Biol. Chem. 275, 23161–23168. 10.1074/jbc.M001448200 10779507

[B19] Korobkova E. A. (2015). Effect of natural polyphenols on CYP metabolism: implications for diseases. Chem. Res. Toxicol. 28, 1359–1390. 10.1021/acs.chemrestox.5b00121 26042469

[B20] Kullak-Ublick G. A. Ismair M. G. Stieger B. Landmann L. Huber R. Pizzagalli F. (2001). Organic anion-transporting polypeptide B (OATP-B) and its functional comparison with three other OATPs of human liver. Gastroenterology 120, 525–533. 10.1053/gast.2001.21176 11159893

[B21] Lánczky A. Győrffy B. (2021). Web-based survival analysis tool tailored for medical research (KMplot): development and implementation. J. Med. Internet Res. 23 (7), e27633. 10.2196/27633 34309564 PMC8367126

[B22] Le Vee M. Jouan E. Moreau A. Fardel O. (2011). Regulation of drug transporter mRNA expression by interferon-γ in primary human hepatocytes. Fundam. Clin. Pharmacol. 25 (1), 99–103. 10.1111/j.1472-8206.2010.00822.x 20199580

[B23] Li N. Li L. Chen Y. (2018). The identification of core gene expression signature in hepatocellular carcinoma. Oxid. Med. Cell. Longev. 2018, 3478305. 10.1155/2018/3478305 29977454 PMC5994271

[B24] Li T.-T. An J.-X. Xu J.-Y. Tuo B.-G. (2019). Overview of organic anion transporters and organic anion transporter polypeptides and their roles in the liver. World J. Clin. Cases 7 (23), 3915–3933. 10.12998/wjcc.v7.i23.3915 31832394 PMC6906560

[B25] Llovet J. M. Zucman-Rossi J. Pikarsky E. Sangro B. Schwartz M. Sherman M. (2016). Hepatocellular carcinoma. Nat. Rev. Dis. Prim. 2, 16018. 10.1038/nrdp.2016.18 27158749

[B26] Lu Y. Li C. Chen H. Zhong W. (2018). Identification of hub genes and analysis of prognostic values in pancreatic ductal adenocarcinoma by integrated bioinformatics methods. Mol. Biol. Rep. 45 (6), 1799–1807. 10.1007/s11033-018-4325-2 30173393

[B27] Marta M. Ferreira P. G. Reverter F. DeLuca D. S. Monlong J. Sammeth M. (2015). The human transcriptome across tissues and individuals. Research 348, 660–665. 10.1126/science.aaa0355 PMC4547472 25954002

[B28] Michael M. Doherty M. M. (2007). Drug metabolism by tumours: its nature, relevance and therapeutic implications. Expert Opin. Drug Metab. Toxicol. 3, 783–803. 10.1517/17425255.3.6.783 18028025

[B29] Michalski C. Cui Y. Nies A. T. Nuessler A. K. Neuhaus P. Zanger U. M. (2002). A naturally occurring mutation in the SLC21A6 gene causing impaired membrane localization of the hepatocyte uptake transporter. J. Biol. Chem. 277, 43058–43063. 10.1074/jbc.M207735200 12196548

[B30] Nies A. T. Niemi M. Burk O. Winter S. Ulrich M. Z. Stieger B. (2013). Genetics is a major determinant of expression of the human hepatic uptake transporter OATP1B1, but not of OATP1B3 and OATP2B1. Genome Med. 5, 1. 10.1186/gm405 23311897 PMC3706890

[B31] Oyama T. Kagawa N. Kunugita N. Kitagawa K. Ogawa M. Yamaguchi T. (2004). Expression of cytochrome P450 in tumor tissues and its association with cancer development. Front. Biosci. 9, 1967–1976. 10.2741/1378 14977602

[B32] Parikh N. D. Mehta A. S. Singal A. G. Block T. Marrero J. A. Lok A. S. (2020). Biomarkers for the early detection of hepatocellular carcinoma. Cancer Epidemiol. Biomarkers Prev. 29 (12), 2495–2503. 10.1158/1055-9965.EPI-20-0005 32238405 PMC7529652

[B33] Ren X. Ji Y. Jiang X. Qi X. (2018). Downregulation of CYP2A6 and CYP2C8 in tumor tissues is linked to worse overall survival and recurrence-free survival from hepatocellular carcinoma. Biomed. Res. Int. 2018, 5859415. 10.1155/2018/5859415 30148168 PMC6083600

[B34] Sanchez-Vicente L. Morente-Carrasco A. Marin J. J. G. Briz O. Muñoz-Bellvís L. Sanchez-Vicente L. (2023). Relevance of the organic anion transporting polypeptide 1B3 (OATP1B3) in the personalized pharmacological treatment of hepatocellular carcinoma. Biochem. Pharmacol. 214, 115681. 10.1016/j.bcp.2023.115681 37429423

[B35] Savannah J. M. F. Wu L. Tasha K. Unadkat J. (2019). Organic anion transporting polypeptide 2B1 - more than a glass-full of drug interactions. Pharmacol. Ther. 196, 204–215. 10.1016/j.pharmthera.2018.12.009 30557631

[B36] Schulte R. R. Ho R. H. (2019). Organic anion transporting polypeptides: emerging roles in cancer pharmacology. Pharmacology 95 (5), 490–506. 10.1124/mol.118.114314 PMC6442320 30782852

[B37] Sheweita S. A. (2000). Drug-metabolizing enzymes: mechanisms and functions. Curr. Drug Metab. 1, 107–132. 10.2174/1389200003339117 11465078

[B38] Szklarczyk D. Franceschini A. Wyder S. Forslund K. Heller D. Huerta-Cepas J. (2014). STRING v10: protein–protein interaction networks, integrated over the tree of life. Nucleic Acids Res. 43 (D1), 447–452. 10.1093/nar/gku1003 PMC4383874 25352553

[B39] Thakkar N. Slizgi J. R. Brouwer K. L. R. (2017). Effect of liver disease on hepatic transporter expression and function. J. Pharm. Sci. 106 (9), 2282–2294. 10.1016/j.xphs.2017.04.053 28465155 PMC5614511

[B40] TianranH.TsangY. P.YinM.MaoQ.JashvantD. (2024). Dysregulation of human hepatic drug transporters by proinflammatory cytokines. J. Pharmacol. Exp. Ther. 391 (1), 82–90. 10.1124/jpet.123.002019 39103232

[B41] Todd S. R. Douglas H. Chand Nisha M. Shiffman S. R. K. Luketic M. L. (2008). Surveillance for hepatocellular carcinoma in patients with cirrhosis improves outcome. Am. J. Med. 212, 119–126. 10.1016/j.amjmed.2007.09.020 18261500

[B42] Vasuri F. Golfieri R. Fiorentino M. Capizzi E. Renzulli M. Pinna A. D. (2011). OATP 1B1/1B3 expression in hepatocellular carcinomas treated with orthotopic liver transplantation. Virchows Arch. 459, 141–146. 10.1007/s00428-011-1099-5 21691816

[B43] Villanueva A. (2019). Hepatocellular carcinoma. N. Engl. J. Med. 380 (15), 1450–1462. 10.1056/NEJMra1713263 30970190

[B44] Wen J. Zhao M. (2021). OATP1B1 plays an important role in the transport and treatment efficacy of Sorafenib in hepatocellular carcinoma. Dis. Markers 2021, 9711179. 10.1155/2021/9711179 34721737 PMC8550862

[B45] Wlcek K. Svoboda M. Riha J. Zakaria S. Olszewski U. Dvorak Z. (2011). The analysis of organic anion transporting polypeptide (OATP) mRNA and protein patterns in primary and metastatic liver cancer. Cancer Biol. Ther. 11 (9), 801–811. 10.4161/cbt.11.9.15176 21383546

[B46] Yan T. Lu L. Xie C. Chen J. Peng X. Zhu L. (2015). Severely impaired and dysregulated cytochrome P450 expression and activities in hepatocellular carcinoma: implications for personalized treatment in patients. Mol. Cancer Ther. 14 (12), 2874–2886. 10.1158/1535-7163.MCT-15-0274 26516155 PMC4674380

[B47] Zhang L. Wang Q. Han Y. Huang Y. Chen T. Guo X. (2023). OSppc: a web server for online survival analysis using proteome of pan-cancers. J. Proteomics 273, 104810. 10.1016/j.jprot.2022.104810 36587732

[B48] Zhu M. Rovella V. Scimeca M. Mauriello A. Shi Y. Bischof J. (2024). Genomic and transcriptomic profiling of hepatocellular carcinoma reveals a rare molecular subtype. Discov. Oncol. 15, 10. 10.1007/s12672-023-00850-9 38228856 PMC10792141

